# Recruiting hard-to-reach populations via respondent driven sampling for mobile phone surveys in Colombia: a qualitative study

**DOI:** 10.1080/16549716.2023.2297886

**Published:** 2024-01-11

**Authors:** Camila Solorzano-Barrera, Mariana Rodriguez-Patarroyo, Angélica Tórres-Quintero, Deivis Nicolas Guzman-Tordecilla, Aixa Natalia Franco-Rodriguez, Vidhi Maniar, Prakriti Shrestha, Andrés I. Vecino-Ortiz, George W. Pariyo, Dustin G. Gibson, Joseph Ali

**Affiliations:** a Institute of Public Health, Pontificia Universidad Javeriana, Bogota, Colombia; b Department of International Health, Johns Hopkins University Bloomberg School of Public Health, Baltimore, MD, USA; c Berman Institute of Bioethics, Johns Hopkins University, Baltimore, MD, USA

**Keywords:** Mobile phone surveys, respondent driven sampling, hard-to-reach population, noncommunicable diseases, older adults, mhealth, inclusion

## Abstract

**Background:**

Uptake of mobile phone surveys (MPS) is increasing in many low- and middle-income countries, particularly within the context of data collection on non-communicable diseases (NCDs) behavioural risk factors. One barrier to collecting representative data through MPS is capturing data from older participants.

Respondent driven sampling (RDS) consists of chain-referral strategies where existing study subjects recruit follow-up participants purposively based on predefined eligibility criteria. Adapting RDS strategies to MPS efforts could, theoretically, yield higher rates of participation for that age group.

**Objective:**

To investigate factors that influence the perceived acceptability of a RDS recruitment method for MPS involving people over 45 years of age living in Colombia.

**Methods:**

An MPS recruitment strategy deploying RDS techniques was piloted to increase participation of older populations. We conducted a qualitative study that drew from surveys with open and closed-ended items, semi-structured interviews for feedback, and focus group discussions to explore perceptions of the strategy and barriers to its application amongst MPS participants.

**Results:**

The strategy’s success is affected by factors such as cultural adaptation, institutional credibility and public trust, data protection, and challenges with mobile phone technology. These factors are relevant to individuals’ willingness to facilitate RDS efforts targeting hard-to-reach people. Recruitment strategies are valuable in part because hard-to-reach populations are often most accessible through their contacts within their social network who can serve as trust liaisons and drive engagement.

**Conclusions:**

These findings may inform future studies where similar interventions are being considered to improve access to mobile phone-based data collection amongst hard-to-reach groups.

## Introduction

Recent studies show that, with some limitations, mobile phone surveys (MPS) can complement face-to-face surveys, allowing for timely data collection in low- and middle-income countries (LMICs) [1–3]. Globally, MPSs face data collection challenges, such as those linked to the geographic reach of mobile networks, variability in adoption and use of mobile phones across demographic groups, and other general technological literacy issues. These challenges have revealed that certain populations can be particularly hard-to-reach—e.g. older adults, rural populations, and people with disabilities, among others. However, these groups tend to also be significantly affected by non-communicable diseases (NCDs), and thus their inclusion in efforts to surveil NCD burdens and risk factors is critical [4,5].

In general, when used to collect information about the public, surveys should be appropriately contextualised and accompanied by recruitment strategies that could potentially increase participation of hard-to-reach populations [6,7]. Qualitative and quantitative studies can strive to do so in part by implementing remote and in-person recruitment strategies such as respondent-driven sampling (RDS), snowball sampling [8], and chain-referral/social network recruitment [9] via email, personal, and mobile interactions [5].

Studies applied RDS as a plausible face-to-face recruitment method for sex workers, substance users, and people with undiagnosed or untreated health conditions, among other vulnerable hidden groups [9–11]. Limited literature exists, however, on implementing RDS through mobile phones to strategically recruit hard-to-reach populations [7,12,13]. To our knowledge, this is the first implementation research study conducted in a Latin American middle-income country that investigated the potential of RDS techniques applied through MPS to access hard-to-reach populations with automated Interactive Voice Response (IVR) surveys and Computer-Assisted Telephone Interviews (CATI).

This qualitative study accompanied the deployment of a ‘parent’ mobile phone survey of NCD risk-factors across Colombia. The principal research question that motivated our parent study was as follows: What are the necessary conditions for mobile phone surveys to become valid, feasible and high-quality mechanisms for NCD risk factor monitoring in low- and middle-income countries? Concurrently, another MPS piloted RDS techniques to increase the participation of a hard-to-reach population (those over 45 years old) and collected basic demographic details from potential seed participants.

The present qualitative study investigated the factors that influenced the perceived acceptability of a RDS recruitment method for MPS involving people over 45 years of age living in Colombia. The age was set to 45 years and over based on prior research [14,15] that addressed the difficulty to recruit individuals in the two oldest age groups (45–59, 60+ years). The findings reported here reveal the implementation experiences of those involved in effectuating the RDS recruitment component – both members of call centres and call recipients who were asked, through an automated survey or by live call centre operators to facilitate the sampling approach.

## Methods

### Study design

Following the integration of RDS techniques (used interchangeably with snowball sampling in this context) into parent IVR and CATI mobile phone surveys, three qualitative data collection methods [16–18] were used as the principal data collection methods for our study: focus group discussions (FGDs), semi-structured interviews for feedback, and surveys with open and closed-ended items. All data were collected from January to May 2021.

### Recruitment approach and sampling

#### Mobile phone surveys

We developed IVR and CATI surveys in Colombia during the COVID-19 pandemic. This survey included an informed consent adapted from previous MPS studies [19–22], and with language that adhered to the Law 1581 of 2012 on data management and protection in Colombia [23]. Recruitment was done using random-digit-dialling [24] and we modelled the four age-groups from the WHO’s STEPwise Approach to Surveillance of NCD risk factors [25,26]. Details on the design and implementation of the MPS parent study can be further explored in a prior publication [27]. Another MPS applied RDS techniques to increase participation of the hard-to-reach population (those over 45 years old) via a first contact with an intermediary family member (hereafter referred to as the ‘seed’).

To operationalise the RDS approach, during the first contact MPS we determined the potential seed’s eligibility to refer an older family member based on the following criteria: 1) being at least 18 years old and 2) having at least two people over 45 years old living within their household, including the ‘seed’. For the IVR survey modality, we offered a call-in phone number to all eligible ‘seeds’ who consented to participate. The number was provided through both a pre-recorded message and via SMS to share with the referral candidate (person over age 45). We asked ‘seeds’ to invite their referral candidates to call the toll-free number and complete the NCD behavioural risk factor survey. Upon NCD survey completion, only the referred relative received an airtime incentive of COP 5000 to their prepaid mobile phones. Seeds did not receive any type of monetary incentive for participating in the RDS approach or for completing the seed survey.

For the CATI survey, the research team applied the following RDS strategies: 1) share a Colombian toll-free number (01800) with referral candidate; 2) pass the phone to referral candidate immediately (if available); 3) share a mobile number with referral candidate to call and receive an immediate call back from a call centre agent; and 4) reschedule the call based on referral candidate’s availability. We offered ‘seeds’ each strategy in a tiered approach, until the last month when they could choose any of the four options they preferred.

The IVR survey implemented only strategy #3, where the ‘seed’ provided the referral candidate with a phone number to call and receive an immediate call back from a call centre agent. In both IVR and CATI, periodic follow-up SMS messages were sent to the ‘seeds’ with a reminder message that encouraged them to share the mobile phone number with a referral candidate.

### Data collection materials, participants & process

#### Seed survey

Around 1 week after the RDS MPS survey, the research team called the ‘seeds’ to conduct a brief ‘seed’ survey. Call time windows were established in two- or three-hour periods (morning, afternoon, and early evening). The team identified whether the ‘seed’ shared the number with the referral candidate and inquired about this decision and the perceived acceptability of the RDS strategy. Interviewers administered the survey exclusively to ‘seeds’ and registered responses over telephone using a free open-source tool to facilitate mobile data entry (KoBoToolbox) [28].

To explore the factors that influenced the perceived acceptability of the recruitment strategy, we structured the survey into the following categories, established *a priori* by the study team: 1) motives or decision factors of whether to share the number; 2) sentiment towards the data protection law and any perceived future implications for allowing a third party to use personal data; and 3) overall likeability of the RDS strategy based on rating and feedback.

**Figure 1.**
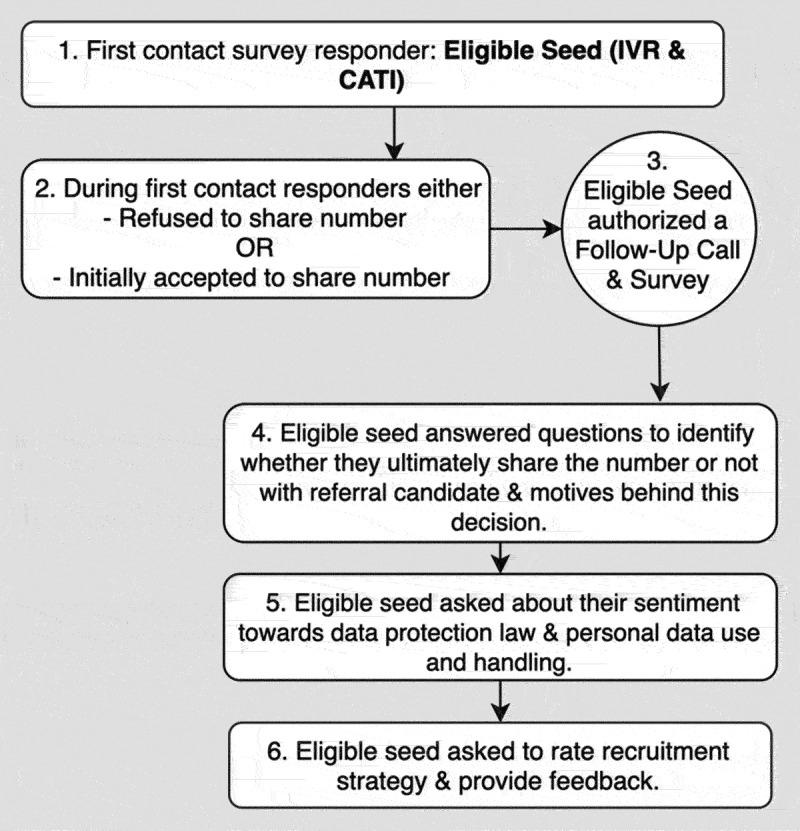
Survey response pathway of seed respondents.

Figure 1 illustrates the possible pathway that a respondent could undergo to be eligible to answer the seed survey and the factors used to investigate perceived acceptability.

#### Focus group discussions and semi-structured interviews

We conducted three FGDs with the call centre agents that administered the CATI surveys. We chose to apply FGDs based on the value attained from this technique as described by several authors in prior works that FGDs enable participants to dialogue, extend, and challenge their perspectives about the topics in question amongst one another [16,18,29]. The agents provided input on the ‘seeds’ receptiveness towards the survey’s consent process and the RDS strategies.

We interviewed the call centre leads via semi-structured interviews during the implementation period and following its completion to receive feedback and better understand how to improve the acceptability of the RDS strategies within the Colombian context. These participants were selected based on their key roles in the CATI survey implementation process. The call centre coordinator and chief operating officer built the CATI survey onto their platform and tested it prior to study launch. The coordinator sent weekly reports detailing numbers dialled, dropped, refused consent, and ‘seeds’ retained and surveyed, among other variables collected throughout the study.

The semi-structured interviews and FGDs were conducted in Spanish via a virtual platform, Zoom; all participants (approximately 6–10 people) agreed to be audio recorded. Using contrast and evaluative questions [16], we sought to better understand the perceived acceptability of the RDS strategy from the perspective of the call agents and call centre leads. Through these conversations, we gained insight on whether they believed the sampling strategies were effective and why or why not; their opinions about the ‘seeds’ sentiments toward data protection; and their suggestions on how to improve the recruitment strategy and its user-friendliness.

**Figure 2.**
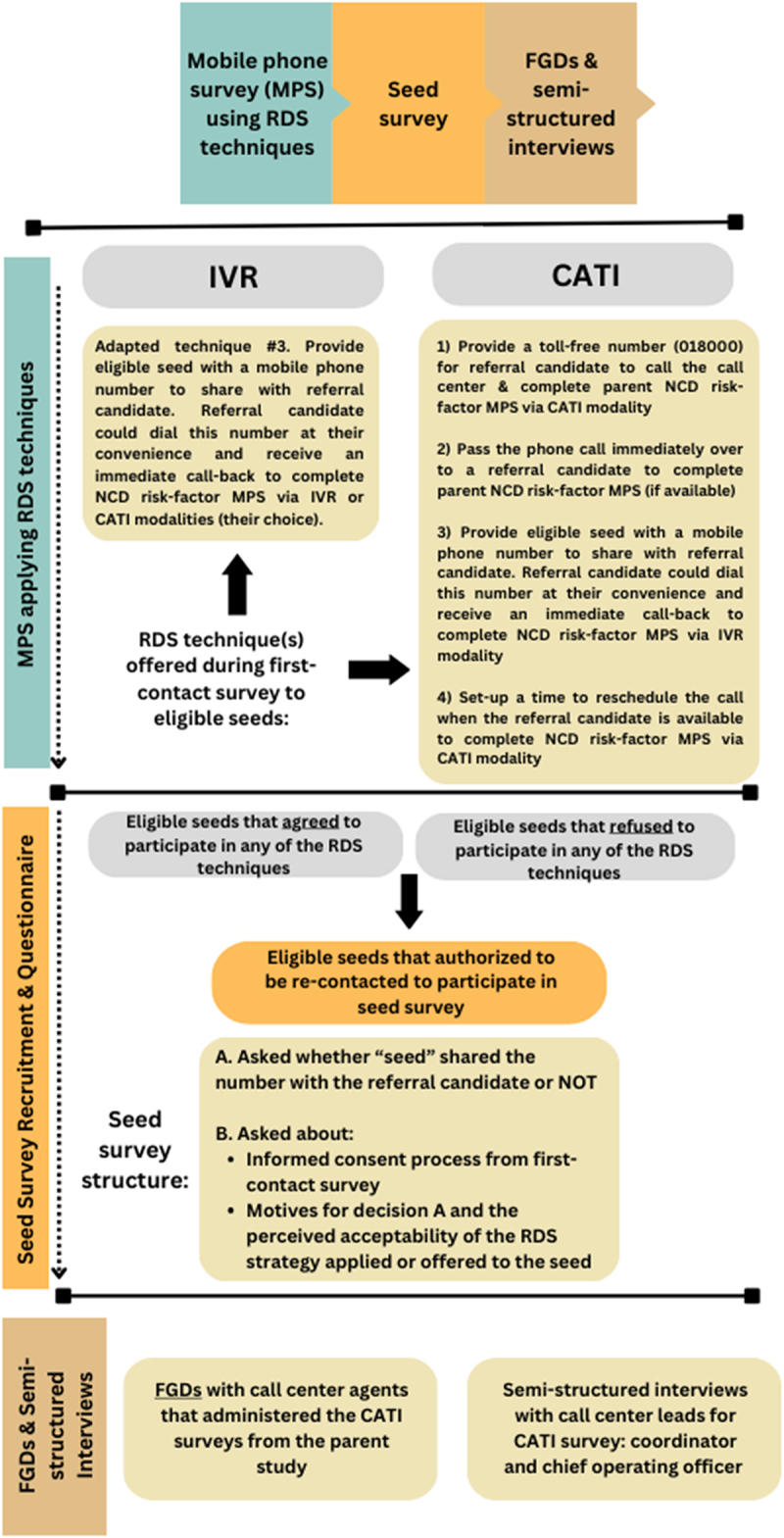
Study design stages and qualitative data collection methods.

The authors adhered to the consolidated criteria for reporting qualitative studies (COREQ) checklist [30] (see supplementary file 1). Figure 2 provides an overview of the complete study design in stages by the type of MPS survey administered and modality, as well as the qualitative data collection methods applied.

### Data management & analysis

#### Seed survey

For open-ended survey items, we coded qualitative findings into four domains. Quantitative results from the closed-ended seed survey questions as well as any data from the referral candidates are not within the scope of this qualitative paper and thus excluded. We tabulated descriptive data on the demographic characteristics of the seed respondents and the outcome numbers from the seeds’ participation in the study.

#### Focus group discussions and semi-structured interviews

Semi-structured interviews [31] and FGD recordings were transcribed in Spanish. The transcripts were thematically coded independently, according to analysis categories established *a priori* by the study team.

The narratives from the participants were grouped to find consensus in shared views and dissent among the different types of participants. To illustrate the findings, representative quotations were included within each thematic area. The coding process, analysis, and results were developed and discussed among the authors to improve the reliability of the findings.

#### Reflexivity

We engaged in a reflexivity practice to evaluate and acknowledge the subjectivity of our role as researchers throughout the course of this study [32]. The research team participated in a focus group discussion involving our principal investigator, qualitative co-investigator, field coordinator, and research assistant to unpack how our reflections and familiarity with the context might have shaped the orientation of this research [33] and the transferability of research for future studies [34,35]. We debriefed with colleagues abroad during informal conversations and formal meetings to react to unforeseen circumstances that required us to adapt the recruitment strategy and make methodological decisions based on the incoming data, which sought to strengthen the credibility and dependability of our methodological approach [34].

Given our prior understanding and familiarity with the Colombian social and cultural environment — such as the perceived insecurity of receiving a call from an unknown number or dialling out to a previously unknown number, and the recent introduction of national data protection regulations that have received significant public attention — this facilitated our ability to explore and apply more suitable RDS techniques throughout the study. Reflexivity was a key tool for analysing how socio-cultural and legal differences between countries represent methodological challenges, especially for those countries where personal data protection laws are more robust. We also recognised the relevance of incorporating sociocultural differences in adapting the instruments to the contexts of use.

#### Ethical considerations

This study was approved by the Research and Ethics Committee of the Public Health Institute of Pontificia Universidad Javeriana (0001 from 2020) and the Johns Hopkins Bloomberg School of Public Health Institutional Review Board (#00007318).

‘Seed’ interviews were administered via a survey structure only to those ‘seeds’ who had consented (verbally for CATI or via a keypad response for IVR) to be re-contacted for additional data collection. The opening survey script informed the responder of the study’s objectives and purpose. The interviewer obtained verbal consent from the interviewee before proceeding. During data collection and entry, each mobile phone number was anonymised by assigning it a unique ID to keep the participants’ personal information protected and confidential. All interview and FGD participants provided their informed consent to participate and granted permission to have the discussions audio recorded.

## Results

The study population involved the following groups as shown in Table 1.

**Table 1.** Study population.

Population	n (people)	Data collection instrument
Eligible seeds (IVR and CATI) who *agreed* to refer candidates and completed ‘seed’ survey	115	‘Seed’ survey – open and closed-ended items
Eligible seeds (IVR and CATI) who *refused* to refer candidates and completed ‘seed’ survey	17	‘Seed’ survey – open and closed-ended items
Call centre agents that administered CATI first contact surveys	10	Focus group discussions
Chief operating officer and call centre coordinator	2	Semi-structured interviews

In IVR, the MPS study that collected demographic data and applied the RDS techniques to seeds was administered for one-month in 2021. Within that period, 386 of the people that responded to the RDS question and met the inclusion criteria were categorised as eligible seeds. During the three-month CATI implementation period of the MPS study, 442 people were classified as eligible seeds and offered to partake in the RDS technique. For further details and context on the demographic characteristics of the seed respondents collected from the first contact MPS, refer to the corresponding table in supplemental file 2.

Table 2 summarises the number of seeds contacted, declined, and completed seed surveys. Seed survey administration was purposely kept concise, lasting 5 min on average.

**Table 2.** Number of seeds contacted, declined, and completed seed surveys.

		IVR	CATI
Eligible seeds that were offered to participate in RDS technique (first-contact MPS)		386	442
Eligible seeds that refused to partake in RDS technique		118	163
	Seeds that authorised re-contact for seed survey	31	12
	Calls dialed	31	12
	Seed surveys completed	13	4
Eligible seeds that agreed to partake in RDS technique		243	196
	Seeds that authorised re-contact for seed survey	189	182
	Calls dialed	158	152
	Seed surveys completed	61	54

The semi-structured interviews and FGDs lasted between 1 and 2 hours each. The qualitative analysis triangulates all the study groups and three data collection methods to identify factors that contributed to the perceived acceptability of the RDS sampling strategy in Colombia. Ultimately, the information obtained from three techniques (feedback interviews, FGDs, ‘seed’ surveys) were triangulated as criteria for the quality and reliability of the data as suggested by key authors [34,36]. The combined emerging themes that resulted from open and axial coding [16] of the qualitative analysis were the following: 1) Adaptation to cultural context; 2) Trust as a guiding principle in participating; 3) Credibility of administering institution; 4) Comfort with the recruitment strategy; 5) Perceptions of data protection measures; 6) Profile of the call centre agent for CATI; and 7) Knowledge and skills of the target population using mobile phone devices.

### Adaptation to cultural context

Daily practices and collective imaginations regarding calling certain mobile phone numbers influenced the results obtained. For CATI, the ‘seeds’ asked to share a 018000 (toll-free) telephone number with their family member emerged as the group with the lowest referrals. It appeared that various factors contributed to this, including a perception that calling the toll-free number, a landline, from a mobile device would be unusual, infeasible, or potentially costly (despite being labelled toll-free). A FGD participant indicated: ‘*first, we are usually not accustomed to making calls to 018000 lines in Colombia; second, we fear calling that is supposedly free, but then could possibly charge us; third, (…) some of those lines are even blocked by mobile phone* [operators]’ (CATI-FGD7-15).

### Trust as a guiding principle in participating

The perception of security and trust are framed within the Colombian context [21] where situations of telephone extortion are common. For example, a woman who had accepted a phone call during the first contact, but ultimately did not refer her family member, said ‘*For many people [the strategy] can generate distrust. People [in Colombia] have a lot of distrust at this time* [during the COVID-19 pandemic] *… Do you think we are going to share our information?*’ (CATI- Female-249).

Furthermore, when the call centre agents explained the strategy, some perceived mistrust from the ‘seed’, and some agents received insults. One of the agents indicated: ‘*the person does not even allow the entire initial greeting to be completed and instead interrupts “ahh, what jail are you calling me from? Are you going to scam me?*”’ (CATI-FGD1-02). Meanwhile, others commented that the calls were welcomed, and ‘seeds’ showed interest in participating. One of the agents stated, ‘*There are people who are very grateful, people who say*, “*how cool, I felt very good while responding the survey*”’ (CATI-FGD1-03).

Other people considered that the strategy was acceptable because it leveraged trust within family members. A woman noted ‘[having a family intermediary] *provides security, it filters out unwanted calls. You receive the number from someone you know. Also, the respondents call voluntarily, and it is not invasive*’ (IVR- Female-432).

### Credibility of administering institution

Given the context that the study’s data collection occurred during strict isolation and quarantine measures enforced due to the COVID-19 pandemic, people were generally sensitive to health-related matters, and significantly more public health-related messages, of variable quality, were being communicated to the public. As such, some ‘seeds’ expressed a degree of health communication suspicion about the calls and RDS process. On the other hand, for eligible ‘seeds’ who ultimately shared the contact number with their relatives, a factor that contributed to the acceptability of the strategy was institutional confidence.

Institutional trust does not necessarily imply that people must specifically know those implementing the RDS strategy. It appears to be sufficient that the institution generally has a credible reputation. That is, the trust of those who accept the strategy is deposited in the academic institution. A woman noted, ‘[I shared the number] *because whoever called was from the university and the reality is that* [due to the pandemic] *… I think that* [they] *could give me more health-related knowledge*’ (IVR-Female-409).

‘Stay at home’ orders were established in Colombia during the pandemic, which distanced people from in-person health facilities, and contributed to several eligible ‘seeds’ deciding to share the call-in number with their family members because they perceived mobile phone health surveys from the administering institution as useful, interesting, and timely. They felt that the purpose of the survey was beneficial to public health. A male indicated, ‘*I sincerely believe that in these current conditions* [quarantine/isolation], *this is the fastest and most effective way to collect information’* (IVR-Male-269). These responses demonstrated that there were seeds that felt their engagement in the recruitment strategy and the information they provided could facilitate valuable input towards generating public policies.

### Comfort with the recruitment strategy

Lack of understanding of the RDS recruitment approach was reported to decrease its acceptability, while also serving as valuable input to identify potential areas of improvement. For example, a woman who initially had agreed to pass along the call-in number shared that she ultimately decided not to because, ‘*I didn’t believe* [in the strategy], *because* [the voice operator] *didn’t want to ask me the survey although I meet the age requirement*’ (IVR- Female-396). Despite an apparent initial willingness to participate in the survey, the respondent chose not to partake if her role was to be a link to reach the final target respondent.

We found that eligible ‘seeds’ who refused to refer a candidate, demonstrated the need to clearly communicate how to apply the RDS strategy and its purpose. For example, a woman said, ‘*I did not understand the method; I was taking the survey, why do I have to involve my family? Why call someone else?*’ (IVR-Female-316). The interviewer explained to the potential ‘seed’ the purpose and value of their intermediary role and how this recruitment strategy aimed to increase the NCD risk factor survey response rate for people over 45 years old. Following the explanation, the ‘seed’ acknowledged that she now understood the recruitment strategy; nonetheless, she reiterated her decision not to involve her husband (the eligible referral candidate), as to not put him in a position where he might feel obliged to answer the survey. Instead, the ‘seed’ proposed that the University call him directly just as she had been contacted. This sort of feedback received from ‘seeds’ was also reiterated from call centre agents during the FGDs, which helped the study team identify an area for potential enhancement to the inclusiveness of the recruitment approach. That is, requesting the phone number from the ‘seed’ and calling the relatives directly.

### Perceptions of data protection measures

Colombian data protection laws now require that data owners express their authorisation for processing personal data before answering the survey or that they know that by unequivocal actions they are granting the authorisation, where silence is never understood as approval. However, this also means that certain types of data collection activities, including those conducted over mobile phones, must include detailed and somewhat complicated disclosures about how data will or will not be used. Call centre agents found that in many cases, when people did not understand the effects or implications of authorising the handling of their personal data, those people preferred to end the call. As stated by an agent, ‘[the terms] *can be confusing for people and that when a person feels confused or stunned, they rather hang up. The person feels uncomfortable by this type of question and tends to react coming more from an attitude of feeling rejection*’ (CATI-FGD7-11).

For others, the provided data use disclosures and authorisation element contributed to the acceptability of the recruitment method. A woman noted, ‘*it is good that they ask you* [for authorization] *because if I don’t want to continue the call, it’s bad practice they don’t ask*’ (CATI-Female-38). However, even with the data protection information and consent included, some respondents recognised that nefarious actors might use this aspect to gain false confidence. In this regard, a man indicated, ‘*the truth is that with the country’s situation* [insecurity], *no matter how much authorization they request, one no longer knows who to trust*’ (IVR-Male-260).

### Profile of the call centre agent for CATI

The acceptability of the RDS strategy seemed to increase when call centre agents presented a friendly tone and veered off-script, initiating the call by asking ‘*Do you have a few minutes? I want to give you some information; can I tell you what this survey is about, and you can tell me if you are interested?*’ (CATI-FGD1-12).

Specifically, a researcher highlighted the importance of empathy that call centre agents must convey when applying the RDS strategy, ‘[who is on the other side of] *the mobile line is a human being, (…) with all their needs, suffering, pains, anguish and therefore relating to the other as a human being and not as a commercial client is very important in this research*’ (CATI-FGD4-13). The call centre coordinator indicated that it was common for the agents to have more experience conducting calls and surveys related to commercial sales rather than surveying for applied health research.

### Knowledge and skills of the target population using mobile phone devices

Some ‘seeds’ that received the IVR survey experienced challenges with mobile technology. This was indicated by a woman: ‘*I am quite clumsy with these complicated technological systems, it was* [too] *big* [a task] *for me (…) I do not know what to say, do not trust everything I did, it was more pressing the mobile phone pad, it was a disaster*’ (IVR- Female-316). Another woman added: ‘*I consider that the population they want to reach and in the socioeconomic level we live, don’t use technology and a call from an unknown number generates distrust*’ (CATI- Female-215). The acceptability of the strategy seemed to diminish as more ‘seeds’ responded that their family member, the eligible referral candidate, would not participate. ‘seeds’ considered that their family member would not have time to complete the survey. Others reiterated the belief that older people do not have sufficient technological and cognitive skills required to answer a mobile phone survey. A woman noted: ‘*they* [those over 45 years old] *do not have the initiative to call by themselves. The best thing is to call them directly*. [For me] *it is difficult to explain to those over 45 years old the objective of the survey, and if they do not understand the survey’s objective, they will not call*’ (CATI-Female-53).

Table 3 summarises the overall findings and key recommendations for future mobile phone-based RDS applications.

**Table 3.** Key findings and recommendations relevant to future mobile phone-based RDS.

Factors	CATI	IVR	Improvements for future phone based RDS applications
Adaptation to cultural context	Dialing a toll-free line from a mobile phone is uncommon and appears infeasible or potentially costly for respondent.		Provide a mobile phone number versus land-line for the call-back and ensure the IVR voice is amicable, slow, and clear. Reiterate instructions on how to dial a toll-free number from a cell phone and clarify there is no cost upon dialing. Periodic reflexive analysis and peer debriefing to adapt the MPS instrument based on evolving socio-political contexts.
Trust as a guiding principle in participating	Telephone extortion is common, adding to seeds perceived mistrust upon receiving the first contact call and being asked to engage the referral contact. Yet, building trust with the seed could leverage trust within family members and decrease perceived security threats.		Provide clarity of the institution calling during introduction Offer additional sources that any seed could reference to validate study details, its objectives, and any related institutional affiliations (e.g. sponsor contact information, project webpage, etc.).
Credibility of administering institution	For seeds that refused to refer, perceived health communication suspicion by the seeds about the calls and RDS strategy. For seeds that referred, institutional confidence and trust established by naming the University were key motives.	Seeds perceived RDS was the quickest and most efficient way to collect data and reach populations over 45 years old. Seeds believed that their participation in the study contributed to future public policies.	Provide resources (i.e. institutional website, informative infographic on the study, institutional contact information) and clearly orient respondents on how to access these resources to validate that indeed it is a credible institution conducting the survey.
Comfort with the recruitment strategy		Disagreement with the RDS approach to exclude seeds from participating also as referral candidates themselves; these seeds opted out of serving as an intermediary.	Modify inclusion criteria to allow for the seed candidate to qualify as the referral candidate (if age requirement met). Consider directly contacting these referral candidates after having received their contact through the seed.
Perceptions of data protection measures	A lack of understanding of the terminology used in the data protection and handling, and misinterpretation of any future implications if they authorised consent of their data. Yet, being asked for consent generated positive sentiment for some seeds.		Leveraging CATI call centre operator to build rapport, answer and remedy any concerns by respondent (more challenging in IVR and preconceived notions of telephone extortion). More piloting and adaptation of the informed consent, specifically the terminology and complexity of language included in the data protection measures.
Profile of the call centre agent for CATI	Call centre agents’ tone and amicability (i.e. greet with respect and warmth) while engaging with potential seeds enhanced perceived acceptability of seed’s willingness to participate in the RDS technique. Seeds were deterred by call centre agents who had more commercial experience in sales versus health research experience.		Contracting call centre agents with experience working with academic institutions or conducting health-related research. Narrowing the selection criteria when recruiting future staff to administer CATI surveys. Provide further training and educational or support resources on the health context of the survey being administered. More mock simulations of potential interactions with seeds (positive and negative ones as practice).
Knowledge and skills of the target population using mobile phone devices		Seeds experienced technological challenges interacting with the IVR system and responding correctly on their mobile devices. Seeds challenged the RDS technique’s ability to accurately relay the study’s objective via an intermediary, argued this could further disengage referral candidates of ages 45 years or over.	Streamline the response steps during the IVR survey and provide more clear guidance on maneuvering the survey on the mobile device (e.g. more repeat, help, and attempts before being dropped from the call). Further piloting and additional implementation studies assessing the response rate of seeds and referral candidates using the IVR modality to respond to the surveys.

## Discussion

This study explored the factors that could affect the perceived acceptability of applying RDS techniques to increase participation of Colombians over 45 years old in IVR and CATI NCD risk factor surveys conducted over mobile phones. To our knowledge, this was the first implementation research study conducted in a middle-income country within Latin America to apply RDS as a potential engagement strategy for hard-to-reach populations in MPS, specifically via IVR and CATI surveys.

Our findings suggest that when accounting for certain factors that can contribute to the perceived acceptability of this strategy, RDS techniques can be supportive as a tool to engage hard-to-reach populations (namely mobile phone users over age 45). Specifically, cultural adaptation, public trust, institutional credibility, understanding of the RDS approach, perception of data protection laws, profile of the call centre agent, and ease to apply and overcome challenges with mobile phone technology. Furthermore, some eligible ‘seeds’ did not fully trust the interaction with the call centre agent or others who were first contacted via IVR and were somewhat unfamiliar with health surveys administered via mobile phones. Contextually appropriate language and design features should be incorporated into surveys, and molded during recruitment and enrolment if needed as has been investigated in prior MPS research in Colombia [21]. Strategies for surveying hard-to-reach populations must align within the specific implementation context. According to Firchow & Mac Ginty, the survey development process for these types of populations should include those who are investigated and ensure that local voices are heard [37]. We suggest the same is true for the development of recruitment techniques, especially those that may be novel with respect to their application in a MPS context. Past mHealth implementation science studies in other health-related fields have found that through such participatory efforts and co-developing, the relevance and acceptability of the research and its engagement strategies will increase [38,39].

Our study confirmed that even over the medium of a mobile phone, part of the reason why RDS strategies have added value is because hard-to-reach populations are often most accessible through those who they trust. If willing to engage, ‘seeds’ can serve as trust liaisons, with their social networks and social capital playing a major role in the success of a phone-based outreach campaign [40]. Trust is a determining condition in the acceptability of the RDS recruitment strategy. Once trust is established, this precedent serves as a driving force for the ‘seed’ to share the number and motivate or potentially oblige their family members to partake in the mobile phone survey. Alternatively, if trust is not established, the ‘seed’ could dissuade and hinder the RDS strategy by affecting the referred family member’s willingness to participate in the study. Leighton et al. also showed the viability of using social networks to apply RDS strategies [41].

The performance and experiences of call centre agents who are involved in implementing commercial surveys have been widely examined [42–44], but the same is not the case for when they serve to administer surveys for health research purposes. Nonetheless, our findings coincide with what is reported in the literature about the link between the call centre agent’s emotional intelligence and their performance [45]. Our study found that call centre agents who displayed cordial, off-script, and non-confrontational approaches seemed to retain ‘seeds’ and enhance the overall perceived acceptability of RDS. A challenge in our RDS strategy was its dependence on the ‘seed’ to provide the phone number to the potential respondent. A more favourable approach would be to directly request the referral respondent’s phone number during the call, so they can be called from the call centre or the IVR platform and potentially increase engagement and acceptability.

The data indicated that individuals who recalled being asked to agree to let a third party manage their personal data before proceeding with the survey, demonstrated more positive sentiments and were more willing to participate in the call. These ‘seeds’ valued transparency and understood they had the right to opt-out of the call by not consenting.

However, the findings also suggest a possible relationship between misunderstandings of data protection language and drop-offs during the call, indicating that the authorisation step could act as a barrier to participation in the RDS strategy. Some ‘seeds’ dropped off the call as a response to feeling rejected from misunderstanding the data protection measures and its future implications. Other ‘seeds’ stated that whether the authorisation was solicited or not, this component of the call did not add value or institutional credibility in the ‘seed’s’ decision to believe the call was not a scam or intended for malicious purposes.

While the Colombian Law 1581 of 2012 (*Ley Estatutaria 1581 de 2012*) was formulated in part to align data protection practices in the country with that of some other regions globally, research indicates that there are gaps in this regulatory framework given the increased use of emerging technologies and associated data for public health and other purposes. Some of these emerging technology and data uses may not necessarily be contemplated within existing broad legal frameworks [46]. Until regulations are updated, efforts to comply remain important. As we found in our study, many individuals – perhaps regardless of the stated intention of a mobile-phone based interaction – are mindful of societal shifts that seek to bolster data privacy, confidentiality, and autonomy. This awareness can positively or negatively affect their willingness to respond and encourage other household members to respond to a MPS. Consequently, due consideration should be given to the national regulatory and policy climate prior to implementing mobile phone-based data collection. These considerations could influence the success of a nation-wide data collection effort and could also affect how data is interpreted when making comparisons across countries. This should be analysed in detail for future studies performed in LMICs, with opportunities to strengthen consent and other important survey enrolment processes developed through participatory engagement processes, and with an eye towards increasing their suitability for those who are harder to reach [47].

### Limitations

The MPS that incorporated the RDS feature was implemented over a period of only 4 months. Future studies should consider collecting survey data over a longer period, allowing for a more representative sample of potential and eligible ‘seeds’. Further, the individuals we surveyed were limited to those who had previously provided consent to be re-contacted. As such, the perspectives of individuals who actively declined participation are not reflected in our study, though we did interview several individuals who agreed to speak to us despite not agreeing to serve as a ‘seed’.

Four pragmatic modifications of the RDS method were implemented in CATI (but not in IVR) in an effort to increase the likelihood of its perceived acceptability. Overall, there was a low number (*n* = 4) of completed seed surveys for the CATI group that rejected to participate in any recruitment strategy. As a result, the research team transcribed 49 of the first-contact CATI survey recordings from this group to extract any information available regarding their decision to refuse to partake in the strategy. This transcribed data complemented the qualitative data collection and analysis. For future studies, we suggest establishing a longer implementation period for each RDS strategy in both CATI and IVR modalities. These RDS strategies include using a Colombian toll-free or mobile phone number, ensuring immediate callbacks, or facilitating call transfers to referral candidates. Extending each strategy’s implementation timeframe could yield a more comprehensive comparison by modality, allowing for further assessment of their feasibility, acceptability, and impact on response rates.

## Conclusions

There are several key and possibly generalisable takeaways from our efforts to adapt and implement RDS recruitment processes for a mobile phone health survey in Colombia, to access harder-to-reach people (namely those over 45 years of age). The perceived acceptability of such a strategy depends largely on ethical sensitivity, the regulatory and policy climate, aspects of institutional and procedural trust, technological familiarity, and the call agent’s profile. Also, the default procedure should rely on the call centre to contact the respondent based on the information from the ‘seed’, eliminating reliance on the ‘seed’ to proactively recruit. These factors should be considered during the design and implementation of future strategies that seek to engage hard-to-reach populations through RDS techniques over mobile phones.

## Supplementary Material

Supplemental Material

## Paper context

The findings from phase I and II of the parent study’s implementation are published separately [20, 21, 27]. This study presents key steps to enhance the perceived acceptability of employing recruitment strategies, specifically respondent-driven sampling, in conjunction with mobile phone surveys to increase participation of the population most affected by non-communicable diseases in Colombia – people over 45 years of age.
