# Supplementary material for: Recruiting hard-to-reach populations via respondent driven sampling for mobile phone surveys in Colombia: a qualitative study
Source: Glob Health Action. 2024 Jan 11;17(1):2297886. doi: 10.1080/16549716.2023.2297886 (PMC10786427; doi:10.1080/16549716.2023.2297886)
Supplement: Supplemental Material [file ZGHA_A_2297886_SM5624.docx]

**Supplemental online material File 1.Consolidated criteria for reporting qualitative studies (COREQ): 32-item checklist**[48]

| **No. Item** | **Guide questions/description** | **Reported on Page # / Section** |
| --- | --- | --- |
| **Domain 1: Research team and reﬂexivity** |  |  |
| *Personal Characteristics* |  |  |
| 1. Inter viewer/facilitator | Which author/s conducted the interview or focus group? | 5 / “methods” |
| 2. Credentials | What were the researcher’s credentials? E.g. PhD, MD | 42 / Supplemental Online Material: File 2. “Author Biographies” |
| 3. Occupation | What was their occupation at the time of the study? | 42 / Supplemental Online Material: File 2. “Author Biographies” |
| 4. Gender | Was the researcher male or female? | 42 / Supplemental Online Material: File 2. “Author Biographies” |
| 5. Experience and training | What experience or training did the researcher have? | 42 / Supplemental Online Material: File 2. “Author Biographies” |
| *Relationship with participants* |  |  |
| 6. Relationship established | Was a relationship established prior to study commencement? | **No** |
| 7. Participant knowledge of the interviewer | What did the participants know about the researcher? e.g. personal goals, reasons for doing the research | 5-6, 8 / “methods”  **Participants were briefed on the purpose of the study & it’s objectives, the interviewers obtained verbal consent from the interviewee before proceeding.** |
| 8. Interviewer characteristics | What characteristics were reported about the inter viewer/facilitator? e.g. Bias, assumptions, reasons and interests in the research topic | 7-8 / “methods, reflexivity” |

| **Domain 2: study design** |  |  |
| --- | --- | --- |
| *Theoretical framework* |  |  |
| 9. Methodological orientation and Theory | What methodological orientation was stated to underpin the study? e.g., grounded theory, discourse analysis, ethnography, phenomenology, content analysis | 3, 6-7 / “methods, study design; data management & analysis” |
| *Participant selection* |  |  |
| 10. Sampling | How were participants selected? e.g., purposive, convenience, consecutive, snowball | 3-4 / “Methods, Recruitment Approach” |
| 11. Method of approach | How were participants approached? e.g. face-to-face, telephone, mail, email | 3-6 / “Methods, Recruitment Approach” |
| 12. Sample size | How many participants were in the study? | 5 / “Methods, Participants” |
| 13. Non-participation | How many people refused to participate or dropped out? Reasons? | Number not specified / 17 / “Limitations” |
| *Setting* |  |  |
| 14. Setting of data collection | Where was the data collected? e.g. home, clinic, workplace | 5 / “Methods, Data Collection Materials & Process” |
| 15. Presence of non-participants | Was anyone else present besides the participants and researchers? | **No** |
| 16. Description of sample | What are the important characteristics of the sample? e.g. demographic data, date | 4, 13 & 426 /Supplemental file. Table “Demographic characteristics of seed survey respondent by modality”**.** |
| *Data collection* |  |  |
| 17. Interview guide | Were questions, prompts, guides provided by the authors? Was it pilot tested? | 5-6 / “Methods, Data Collection Materials & Process” |
| 18. Repeat interviews | Were repeat interviews carried out? If yes, how many? | **No** |
| 19. Audio/visual recording | Did the research use audio or visual recording to collect the data? | 6 / “Methods, Data Collection Materials & Process” |
| 20. Field notes | Were ﬁeld notes made during and/or after the interview or focus group? | 5-8 / **Both, see**  “Methods” |
| 21. Duration | What was the duration of the interviews or focus group? | 5-6 / “Methods, Data Collection Materials & Process” |
| 22. Data saturation | Was data saturation discussed? | **Yes, not specifically included in manuscript, discussed between local and abroad colleagues, see pgs. 7-8 “Reflexivity”** |
| 23. Transcripts returned | Were transcripts returned to participants for comment and/or correction? | **No** |
| **Domain 3: analysis and ﬁndings** |  |  |
| *Data analysis* |  |  |
| 24. Number of data coders | How many data coders coded the data? | **One (MRP)** |
| 25. Description of the coding tree | Did authors provide a description of the coding tree? | 7/ "Methods" |
| 26. Derivation of themes | Were themes identiﬁed in advance or derived from the data? | 5-7 / “Methods, study design; data management & analysis” |
| 27. Software | What software, if applicable, was used to manage the data? | 6 / “Methods, data management & analysis” |
| 28. Participant checking | Did participants provide feedback on the ﬁndings? | **Yes,** **for participants in the FGDs and feedback interviews, but not directly from seed participants**  5-6 & 8-13 / “Methods” & “Results” |
| *Reporting* |  |  |
| 29. Quotations presented | Were participant quotations presented to illustrate the themes/ﬁndings? Was each quotation identiﬁed? e.g. participant number | 8-14 / “Results” |
| 30. Data and ﬁndings consistent | Was there consistency between the data presented and the ﬁndings? | **Yes** |
| 31. Clarity of major themes | Were major themes clearly presented in the ﬁndings? | **Yes** |
| 32. Clarity of minor themes | Is there a description of diverse cases or discussion of minor themes? | **Yes**  14-17 / “Discussion” |

Developed from: Tong A, Sainsbury P, Craig J. Consolidated criteria for reporting qualitative research (COREQ): a 32-item checklist for interviews and focus groups. *International Journal for Quality in Health Care*. 2007. Volume 19, Number 6: pp. 349 – 357, https://doi.org/10.1093/intqhc/mzm042

**Supplemental Online Material: File 2.**

**Demographic characteristics of seed survey respondent by modality**

|  | **CATI** | | | **IVR** | | |  |
| --- | --- | --- | --- | --- | --- | --- | --- |
| **First contact survey** | **Refused to share number % (n)** | **Accepted to share number % (n)** | **N** | **Refused to share number % (n)** | **Accepted to share number % (n)** | **N** |  |
|  |  |  |  |  |  |  |  |
| **Age-group (years)** |  | | | | | |  |
| **18-29** | 34% (10) | 66% (19) | 29 | 21% (4) | 79% (15) | 19 |  |
| **30-44** | 55% (6) | 45% (5) | 11 | 8% (1) | 92% (12) | 13 |  |
| **45-59** | 56% (19) | 47% (15) | 34 | 19% (5) | 81% (22) | 27 |  |
| **60+** | 52% (13) | 48% (12) | 25 | 20% (3) | 80% (12) | 15 |  |
| **Sex** |  | | | | | |  |
| **Female** | 41% (22) | 59% (32) | 54 | 25% (11) | 75% (33) | 44 |  |
| **Male** | 58% (26) | 42% (19) | 45 | 7% (2) | 93% (28) | 30 |  |
| **Geographical Area** |  | | | | | |  |
| **Rural** | 41% (9) | 59% (13) | 22 | 14% (2) | 86% (12) | 14 |  |
| **Urban** | 51% (39) | 49% (38) | 77 | 18% (11) | 82% (49) | 60 |  |
| **Highest Education Level** |  | | | | | |  |
| **No school** | - | 100% (6) | 6 | 18% (2) | 82% (9) | 11 |  |
| **Primary** | 41% (7) | 59% (10) | 17 | 17% (3) | 83% (15) | 18 |  |
| **Secondary** | 45% (14) | 55% (17) | 31 | - | - | - |  |
| **Technical Professional** | 57% (8) | 43% (6) | 14 | 18% (4) | 82% (18) | 22 |  |
| **Undergraduate/ Postgraduate** | 61% (19) | 39% (12) | 31 | 17% (4) | 83% (19) | 23 |  |

**Supplemental Online Material: File 3.**

**Author Biographies**

Camila Solorzano-Barrera, MPH is a project policy analyst at the University of California San Francisco. At the time of data collection for this study, she served as assistant researcher and environmental consultant at the Institute of Public Health, Pontificia Universidad Javeriana in Bogotá, Colombia.

Mariana Rodriguez-Patarroyo, PhD is a researcher at the Institute of Public Health, Pontificia Universidad Javeriana in Bogota, Colombia. The scope of her research is related to socio-environmental conflicts, mHealth and personal data protection, and on the application of qualitative methods for bioethics research.

Angelica Torres-Quintero, PhD is an assistant professor at the Public Health Institute of the Pontificia Universidad Javeriana. She uses qualitative methods to study the perceptions, attitudes, behaviors and social representations associated with risk factors and health vulnerabilities.

Deivis Nicolas Guzman-Tordecilla, MSc, is a current doctoral student within the Department of International Health at Johns Hopkins University Bloomberg School of Public Health. At the time of data collection for this study, he served as field coordinator for the parent mobile phone surveys.

Aixa Natalia Franco-Rodriguez is an anthropologist from the Pontificia Universidad Javeriana. She’s interested in the application and reflection around social problems related to health, and currently works as a Monitoring and Evaluation Assistant at the Norwegian Refugee Council.

Vidhi Maniar, MS, MPH is a project manager at the Johns Hopkins University Bloomberg School of Public Health. Her research focuses on the use of mobile phone surveys to monitor NCD risk factors in Bangladesh, Colombia, Tanzania, and Uganda.

Andres I. Vecino-Ortiz, PhD, MSc is a research scientist at the Johns Hopkins University Bloomberg School of Public Health. As a health economist, his research centers on health systems issues with a special focus on the economics of prevention of NCDs and injuries in Latin America.

George W. Pariyo, PhD, MSc was a senior scientist at the Department of International Health Johns Hopkins University Bloomberg School of Public Health while serving as Principal Investigator during this project’s conceptualization and methodological design. He conducts studies that apply innovative strategies seeking to strengthen health systems.

Dustin G. Gibson, PhD is an associate scientist and Director of Data4Health at Johns Hopkins Bloomberg School of Public Health. He works on the application and evaluation of mobile and digital health technologies to strengthen and improve health systems in lower income countries.

Joseph Ali is associate professor, at the Department of International Health, Johns Hopkins Bloomberg School of Public Health and associate director of Global Programs for the Johns Hopkins Berman Institute of Bioethics. His research involves a range of challenges in domestic and global health ethics, Health Law & Policy, mHealth, and Patient-Centered Research.
